# Anti-PD-1 Therapy Alleviates *Klebsiella pneumoniae*-Induced Liver Abscess by Effectively Activating Kupffer Cells

**DOI:** 10.4014/jmb.2602.02040

**Published:** 2026-10-19

**Authors:** Huaxiu You, Minlie Liang, Houxiong Lin, Ying Liu, Xian Huang, Yuanzhong Gong

**Affiliations:** 1 Department of Infectious Diseases, Nanping First Hospital Affiliated to Fujian Medical University, Nanping, Fujian, P. R. China; 2 Department of Geriatric Medicine, Nanping First Hospital Affiliated to Fujian Medical University, Nanping, Fujian, P. R. China; 3 Department of Infectious Diseases, Ningde Municipal Hospital of Fujian Province, Ningde, Fujian, P. R. China

**Keywords:** *Klebsiella pneumoniae*, Liver abscess, Kupffer cells, PD-1

## Abstract

*Klebsiella pneumoniae*-induced liver abscess (KPLA) is the leading cause of pyogenic liver abscess (LA) worldwide. Hepatic Kupffer cells exert protective effects during KPLA progression; thus, elucidating their protective mechanisms and developing novel therapeutic strategies are imperative. A murine KPLA model was established. Hematoxylineosin (HE) staining and tissue bacterial load were analyzed to verify successful *K. pneumoniae* infection. RT-qPCR, Western blot (WB), and flow cytometry were employed to quantify PD-1 expression in Kupffer cells. PD-1-knockdown Kupffer-cell transfection systems and PD-1-antibody treatment groups were established. Bacterial counts (CFU) and ELISA plus flow cytometry for inflammatory cytokines were used to evaluate the relationship between PD-1 expression and Kupffer-cell bacterial clearance. Finally, the KPLA mouse model was treated with PD-1 antibody to validate the role of PD-1 in LA. In the KPLA mouse model, gross abscess formation was observed, and HE staining revealed disorganized hepatic architecture. PD-1 expression in Kupffer cells was markedly up-regulated, and the proportion of PD-1-positive Kupffer cells increased significantly. *In vitro*, both PD-1 knockdown and PD-1-antibody treatment enhanced Kupffer-cell bacterial clearance, accompanied by elevated TNF-α, IL-6, and IL-12 levels. *In vivo*, the PD-1 antibody effectively treated KPLA. KPLA up-regulates PD-1 expression in Kupffer cells, thereby impairing bacterial clearance. Our findings preliminarily indicate that the anti-PD-1 antibody may have application potential in the prevention or early intervention of KPLA.

## Introduction

*Klebsiella pneumoniae* (*K. pneumoniae*) is a Gram-negative, opportunistic pathogen belonging to the order Enterobacterales that is widely distributed in the environment and the human gastrointestinal tract, and constitutes the leading cause of bacterial liver abscess (LA) [1, 2]. As a clinically important opportunistic pathogen, it causes both community- and hospital-acquired infections, including pneumonia, bacteremia, urinary tract infections, and sepsis [3, 4]. In recent years, the emergence of hypervirulent and multidrug-resistant strains has turned this bacterium into a major global public-health threat [5, 6]. *K. pneumoniae*-induced liver abscess (KPLA) is being reported with increasing frequency in Asia and is becoming a worldwide disease [7]. Consequently, identifying novel and effective therapeutic approaches for KPLA is imperative.

Kupffer cells, the liver-resident macrophages, hold a central role in hepatic homeostasis and disease. Constituting the largest pool of tissue macrophages in the body, these immune cells, positioned within hepatic sinusoids, serve as the “hepatic firewall” by clearing blood-borne bacteria and opsonized particles [8, 9], and modulate the intrahepatic immune microenvironment through cytokine secretion and antigen presentation [10, 11]. Kupffer cells comprise two distinct subsets: liver-resident macrophages (F4/80^+^ CD11b^−^ CD68^+^ cells) and hepatic macrophages derived from circulating monocytes (F4/80+ CD11b+ CD68- cells), which exhibit different characteristics during anti-infectious immunity [12]. Under pathological conditions, aberrant activation of Kupffer cells is intimately linked with multiple liver diseases. For example, in fatty liver disease, Kupffer cells drive inflammation and lipid deposition via the release of pro-inflammatory cytokines [13, 14]. In hepatic fibrosis, Kupffer cells activate hepatic stellate cells through mediators such as TGF-β, promoting excessive extracellular-matrix deposition [15, 16]. Kupffer cells exert protective effects during the development of KPLA [17]. Thus, Kupffer cells constitute both the first line of defense against invading pathogens and a key locus whose dysfunctional responses facilitate infection progression; precise modulation of specific subsets or functional pathways may therefore represent a future therapeutic direction.

Programmed death-1 (PD-1) assumes a pivotal role in balancing protective immunity and immunopathology, homeostasis, and tolerance. However, during chronic infection and cancer, it curtails protective immunity [18]. PD-1 is a major regulator of T-cell responses, expressed on activated T cells, B cells, and Treg cells [19], and typically interacts with its ligands PD-L1 and PD-L2 to trigger downstream signaling. In oncology, immune-checkpoint blockade with anti-PD-1/PD-L1 monoclonal antibodies has revolutionized cancer treatment [18, 19]. In infectious diseases, PD-1 expression on macrophages exerts pathological effects by suppressing innate inflammatory responses in sepsis and impairing phagocytosis in active tuberculosis [20, 21]. Increased PD-1 and PD-L1 expression on Kupffer cells and lymphocyte subsets in acute liver failure (ALF) reduces the bacterial clearance capacity of Kupffer cells during acute hepatic injury[22]. To broaden the therapeutic targeting of this pathway, PD-1 function has been examined in DCs, NK cells, and innate lymphoid cells[23]. Nevertheless, the specific role of this pathway in Kupffer cells during LA remains unknown.

We herein investigated the role and function of Kupffer cells during *K. pneumoniae* infection through *in vitro* and *in vivo* experiments to gain deeper insight into their potential protective effects in KPLA.

## Methods

### Animals and Microorganisms

Eight-week-old male C57BL/6J mice were purchased from Hangzhou Hangsi Biotech Co., Ltd. (China). All mice were housed in a pathogen-free animal facility under a standard 12-h light/12-h dark cycle and allowed free access to food and water. *K. pneumoniae* (bio-74093, BIOBW, China) was obtained from Beijing Biobw Biotechnology Co., Ltd. and cultured overnight in LB medium at 37°C.

### Kupffer Cell Isolation

Under anesthesia, mice were perfused via the portal vein with EDTA buffer to clear blood, and the intact liver was harvested into ice-cold cell-wash solution at 4°C. After removal of the gallbladder and capsule, the liver was minced and incubated with collagenase digestion solution (Beyotime, China) pre-warmed to 37°C and placed into 37°C water for 20 min with gentle agitation. The reaction was terminated by the addition of 3–4 volumes of digestion-stop solution. The digested tissue was filtered through a 75-μm cell strainer to obtain a mixed hepatocyte suspension. The suspension was kept at 4°C and centrifuged at 50 × g for 2 min. The hepatocyte-enriched pellet was discarded. The supernatant was then centrifuged at 35 × g for 5 min at 4°C to obtain the non-parenchymal cell (NPC) fraction. The NPC pellet was resuspended in 33% Percoll (Sigma-Aldrich, USA) and centrifuged at 700 × g for 12 min at room temperature for density-gradient purification. The resulting NPC pellet was washed twice with PBS (Servicebio, China) containing 5% FBS (Gibco, USA) to remove residual medium.

Kupffer cells were subsequently isolated from NPCs by magnetic-activated cell sorting (MACS). Briefly, NPCs were incubated with mouse CD16/32 blocking antibody (BioLegend, USA) for 10 min at room temperature, followed by APC-conjugated anti-mouse F4/80 antibody (BioLegend) for 15 min. After labeling, cells were rinsed once with PBS containing 0.5 % BSA (Beyotime) and 2 mM EDTA. APC-conjugated microbeads (Miltenyi, USA) were then added and incubated for 15 min. The labeled cell suspension was rinsed and passed through a MACS column for primary separation. To obtain higher purity, the positively selected cells were subjected to a second MACS column for further purification.

### Mouse Infection

Experimental mice were randomly allocated to two treatment groups (n = 5 per group): Control group: intraperitoneal injection of an equal volume of PBS; KPLA group: intraperitoneal inoculation with *K. pneumoniae* suspension at 1 × 10^3^ CFU/mL (100 μL/10 g body weight). Simultaneously with bacterial inoculation, mice were intraperitoneally injected with 200 μg anti-PD-1 monoclonal antibody (CP151, BioXCell, USA) or an equivalent amount of mouse IgG2a isotype control antibody (BE0085, BioXCell, USA). Forty-eight hours post-treatment, mice were euthanized, and liver tissue and serum samples were collected for subsequent analyses.

### Cell Transfection

pLKO.1-Puro empty vector (sh-NC) and pLKO.1-PD1 (sh-PD1) were bought by Genepharma (China). Kupffer cells were kept in small dishes until reaching 60–70% confluence, then transfected with the above plasmids with Lipofectamine 3000 (L3000001, Invitrogen, USA). After transfection, 1 μg/mL anti-PD-1 monoclonal antibody and 3 μg/mL PD-L1 recombinant protein (HY-P70633, MCE, USA) were added according to experimental grouping. Cells were cultivated for an additional 48 h and then harvested for subsequent detection.

### Bacterial Load Analysis

Blood samples from mice in each experimental group were processed with sterile PBS, serially diluted, and evenly plated onto solid medium. An appropriate amount of liver tissue was weighed, placed in ice-cold PBS, and homogenized thoroughly with a tissue grinder (Tissuelyser-32, China). The resulting tissue suspensions were serially diluted and plated onto solid medium. Plates were incubated at 37°C for 18–24 h, and colony-forming units (CFU) were enumerated.

### Hematoxylin–Eosin (HE) Staining

Liver specimens were immobilized in 4% paraformaldehyde (Citotest, China), dehydrated through graded ethanol, and embedded in paraffin. Tissue sections were prepared with a microtome, deparaffinized, rehydrated, and stained with routine HE. Pathological features of liver tissue were examined and analyzed with a light microscope (Leica, DEU).

### RT-qPCR

Total RNA was extracted from Kupffer cells with TRIzol reagent (Invitrogen, USA). Complementary DNA was synthesized from 500 ng of total RNA with AMV reverse transcriptase (Promega, USA). qPCR was undertaken to quantify PD-1 mRNA levels in Kupffer cells with GAPDH as the internal reference; the specific primer sequences for the amplified genes are listed in Table 1 is incomplete and needs to be supplemented. The amplification reaction was carried out on an ABI 7500 Real-Time PCR system (Applied Biosystems, USA). 2^−ΔΔCt^ method was applied in calculating relative expression levels.

### Western Blot

Kupffer-cell samples were lysed on ice for 30 min with RIPA lysis buffer containing 1 mM PMSF, followed by centrifugation at 12,000 rpm for 10 min to obtain the protein extract. The supernatant was collected and protein concentration was determined with a BCA kit (Beyotime). After the addition of SDS-PAGE loading buffer (Epizyme Biotech, China), proteins were denatured at 98°C. Separating and stacking gels of the appropriate concentration were prepared to resolve proteins. Following electrophoresis, proteins were transferred to PVDF membranes and blocked. To detect target proteins, membranes were incubated overnight at 4°C with primary antibodies anti-PD-1 (ab214421, Abcam, UK), anti-SHP2 (ab300579, Abcam, UK), anti- phospho SHP2 (ab62322, Abcam, UK); anti-STAT1 (ab239360, Abcam), anti- phospho STAT1 (ab109461, Abcam), anti-NF-kB p65 (ab32536, Abcam), anti-phospho NF-kB p65 (ab76302, Abcam), and anti-GAPDH (ab9485, Abcam, UK). The next day, membranes were rinsed three times with TBST (TBS containing 0.1% Tween-20) and incubated with goat anti-rabbit IgG secondary antibody (ab6721, Abcam) for 60 min at room temperature. Finally, chemiluminescent images were acquired with a ChemiScope instrument (ChemiScope, China).

### Flow Cytometry (FCM)

1 × 10^6^ Kupffer cells were resuspended in PBS and stained for 30 min at 4 °C with the following pre-mixed antibody panel: anti-PD-1-PE (12-9985-82, eBioscience, USA), anti-TNF-α-Percp (M100T12-32A, Sungene, China), anti-IL-6-FITC (11-7061-82, eBioscience), anti-IL-12-PE (554479, BD Biosciences, USA), anti-F4/80- PE (111603, Biolegend, USA), and anti-CLEC4F-FITC (AF2784F, R&D systems, USA). After staining, cells were rinsed three times by centrifugation with wash buffer and kept at 4°C in the dark until analysis on a flow cytometer (Agilent, USA).

### ELISA Assay

After co-culture of Kupffer cells with *K. pneumoniae*, the supernatant was collected. The concentrations of TNF-α (E-EL-M3063, Elabscience, China), IL-6 (E-EL-M0044, Elabscience, China), and IL-12 (E-EL-M3062, Elabscience, China) in the supernatant were determined with ELISA kits.

For mouse blood serum samples, a mouse alanine aminotransferase (ALT) detection kit (C009-2-1, Nanjing Jiancheng Bioengineering Institute, China) and a mouse aspartate aminotransferase (AST) detection kit (C010-2-1, Nanjing Jiancheng Bioengineering Institute, China). The serum levels of ALT and AST were measured.

### Bacterial Killing Assay

Kupffer cells isolated and purified from mouse liver were seeded at 2 × 10^4^ cells per well in 48-well plates. The experimental system consisted of DMEM medium (Gibco, USA) containing 10% autologous serum. Kupffer cells were co-cultivated with *K. pneumoniae* at a ratio of 1:100 under standard culture conditions (37°C, 5% CO_2_). After 60 minutes of incubation, the reaction was immediately terminated by placing the plates on ice. The supernatant was carefully removed, and the cell pellet was lysed with alkaline distilled water (pH = 11.0). The lysate was serially diluted and evenly plated onto solid medium. Following incubation at 37°C for 18 h, the number of viable bacteria was determined by colony counting.

### Phagocytosis Assay

Flow cytometry was used to assess the phagocytic capacity of Kupffer cells against *K. pneumoniae*. Briefly, *K. pneumoniae* was labeled with pHrodo Red Active Ester (Thermo Fisher Scientific, USA) according to the manufacturer's instructions. After the corresponding treatments, the culture supernatant was removed, and pHrodo-labeled *K. pneumoniae* resuspended in DMEM containing 0.01 mg/mL bovine serum albumin (BSA) was added. Cells were incubated at 37°C for 1 h. After incubation, cells were washed twice with PBS, harvested by trypsinization, and the phagocytic capacity of Kupffer cells was evaluated by flow cytometry.

### Statistical Analysis

Statistical analysis was processed with SPSS 26.0 and GraphPad Prism 10.1 software. Data, expressed as mean ± standard deviation. *In vivo* data were obtained from n = 5 mice per group; *in vitro* data were obtained from 3 independent biological replicates (n = 3). Normality of the data was assessed with the Shapiro–Wilk test in SPSS. For comparisons among multiple groups, one-way ANOVA was applied to determine significant differences, followed by Tukey’s multiple comparison test for pairwise comparisons. Differences between the two groups were evaluated with an independent samples *t*-test. A *P*-value < 0.05 was considered statistically significant.

## Results

### KPLA Promotes PD-1 Expression in Kupffer Cells

KPLA results from *K. pneumoniae* colonizing the host gastrointestinal tract, traversing the intestinal barrier, and reaching the liver[24]. First, we established a *K. pneumoniae* infection model in C57BL/6J mice and did bacterial enumeration and histopathological analysis of the liver. Compared with the control group, mice infected with *K. pneumoniae* developed obvious hepatic abscesses (Fig. 1A), and HE staining revealed disorganized liver architecture (Fig. 1B). Bacterial loads in the liver and blood were determined. *K. pneumoniae*–infected mice exhibited higher bacterial burdens, confirming successful bacterial colonization (Fig. 1C and 1D) and further verifying infection-induced LA. In addition, compared with the control group, KPLA mice showed remarkably elevated serum ALT and AST levels, confirming that *K. pneumoniae* infection induced hepatocyte injury (Fig. 1E and 1F). It is indicated that liver-resident macrophages (Kupffer cells) exert protective effects during LA[17]. We therefore isolated Kupffer cells from the mouse liver and assessed PD-1 expression by RT-qPCR and Western blot. PD-1 expression was significantly up-regulated in Kupffer cells from infected mice relative to uninfected controls (Fig. 1G and 1H). FCM further demonstrated a higher proportion of PD-1-positive Kupffer cells in the infected group (Fig. 1I). Taken together, KPLA promotes PD-1 expression in Kupffer cells.

### Kupffer Cells with High PD-1 Expression Exhibit Reduced Bacterial Clearance Capacity

To verify the purity of the isolated cells, double staining for F4/80 and CLEC4F was performed by flow cytometry. The results showed that the proportion of F4/80^+^ CLEC4F^+^ double-positive cells among the isolated cells exceeded 80%, indicating that high-purity Kupffer cells were obtained (Supplementary Fig. S1). To further elucidate the regulatory role of PD-1 signaling in Kupffer-cell function, we established PD-1-knockdown Kupffer cells and verified the knockdown efficiency by Western blot. The results showed that, compared with the sh-NC group, sh-PD-1 cells exhibited a significant reduction in PD-1 protein expression, confirming efficient knockdown (Fig. 2A). On this basis, Kupffer cells were co-cultured with *K. pneumoniae* in the presence or absence of PD-L1 recombinant protein (rPD-L1) and divided into four groups (sh-NC+IgG, sh-PD-1+IgG, sh-NC+rPD-L1, and sh-PD-1+rPD-L1) to evaluate the effect of the PD-1 pathway on Kupffer-cell bactericidal activity. The *in vitro* bacterial-killing assay showed that the bactericidal capacity of Kupffer cells against *K. pneumoniae* was enhanced in the sh-PD-1+IgG group, as evidenced by a notable reduction in viable colony counts, whereas the sh-NC+rPD-L1 group, in which PD-1 signaling was exogenously activated by rPD-L1, exhibited increased bacterial survival compared with the sh-NC+IgG group (Fig. 2B). These findings suggest that activation of the PD-1 signaling pathway effectively suppresses Kupffer-cell bactericidal activity. ELISA assays showed that the levels of the pro-inflammatory cytokines IL-6, IL-12, and TNF-α secreted by Kupffer cells were increased after PD-1 knockdown, whereas this effect was reversed by the addition of rPD-L1 to PD-1-knockdown cells (Fig. 2C-2E). Flow cytometry further confirmed that the proportions of IL-6-, IL-12-, and TNF-α-positive cells were elevated in the sh-PD-1+IgG group, whereas in PD-1-knockdown cells supplemented with rPD-L1, the proportions of these positive cells were lower than those in the sh-PD-1+IgG group (Fig. 2F-2H). Flow cytometric analysis of Kupffer cell phagocytic capacity revealed remarkably enhanced phagocytic activity in the sh-PD-1 group relative to controls; however, supplementation with rPD-L1 reversed this elevation in phagocytosis (Fig. 2I). Previous research has shown that PD-1 deficiency enhances STAT1 and NF-κB p65 phosphorylation, thereby promoting macrophage polarization toward the M1 phenotype [25]. In addition, PD-1 knockdown can suppress the SHP2 pathway and activate the NF-κB pathway, thereby enhancing the osteogenic/odontogenic differentiation capacity of SCAPs[26]. Based on these findings, we further investigated whether PD-1 inhibits bacterial clearance via the SHP2/STAT1/NF-κB signaling pathway. Western blot analysis showed that PD-1 knockdown downregulated p-SHP2/SHP2 expression and upregulated p-STAT1/STAT1 and p-NF-κB p-p65/NF-κB p65 levels; conversely, addition of exogenous rPD-L1 to PD-1-knockdown cells reversed these changes in protein expression (Fig. 2J). These results indicate that PD-1 activates SHP2 to suppress NF-κB and STAT1 signaling, thereby downregulating Kupffer-cell inflammatory cytokine production and bacterial clearance.

Given this data, the reduced bacterial clearance capacity of Kupffer cells is attributable to high PD-1 expression.

### PD-1 Antibody Activates the Bacterial Clearance Capacity of Kupffer Cells

To investigate the regulatory effect of anti-PD-1 antibody on Kupffer-cell function, *K. pneumoniae*-infected Kupffer cells were divided into IgG control, anti-PD-1 treatment, rPD-L1 treatment, and anti-PD-1 combined with rPD-L1 treatment groups. The *in vitro* bacterial-killing assay showed that, compared with the IgG group, the bactericidal activity of Kupffer cells against *K. pneumoniae* was enhanced in the anti-PD-1 group. Cells receiving combined anti-PD-1 plus rPD-L1 treatment exhibited lower bacterial survival than the rPD-L1 group, indicating that anti-PD-1 antibody effectively reversed the rPD-L1-induced immunosuppressive effect and partially restored Kupffer-cell bactericidal function (Fig. 3A). ELISA assays showed that the levels of the pro-inflammatory cytokines IL-6, IL-12, and TNF-α secreted by Kupffer cells were notably increased in the anti-PD-1 group, whereas addition of rPD-L1 reversed this increase (Fig. 3B-3D). Flow cytometry further confirmed that rPD-L1 reversed the anti-PD-1-induced increase in the proportions of IL-6-, IL-12-, and TNF-α-positive Kupffer cells (Fig. 3E-3G). Phagocytosis assays showed that the phagocytic activity of Kupffer cells was enhanced in the anti-PD-1 group, whereas addition of PD-L1 recombinant protein reduced this phagocytic activity (Fig. 3H). Western blot analysis showed that anti-PD-1 treatment downregulated p-SHP2/SHP2 expression and upregulated p-STAT1/STAT1 and p-NF-κB p-p65/NF-κB p65 levels; conversely, addition of exogenous rPD-L1 reversed these changes in protein expression (Fig. 3I). Collectively, PD-1 blockade improves the bacterial clearance rate of Kupffer cells in mice with LA.

### PD-1 Blockade Alleviates KPLA

We next investigated whether *in vivo* blockade of the PD-1/PD-L1 axis could improve Kupffer-cell clearance of *K. pneumoniae* and relieve LA in mice. Infected animals received an anti-PD-1 blocking antibody. Compared with isotype-treated controls, anti-PD-1–treated mice exhibited markedly attenuated hepatic abscess formation (Fig. 4A). HE staining showed that disorganized liver architecture was significantly ameliorated by anti-PD-1 treatment (Fig. 4B), while bacterial burdens in liver and blood were substantially reduced (Fig. 4C and 4D). In addition, serum ALT and AST levels in the anti-PD-1 antibody treatment group were notably reduced (Fig. 4E and 4F). Thus, PD-1 blockade alleviates KPLA by activating the phagocytic and bactericidal functions of Kupffer cells.

## Discussion

LA is a severe hepatic infectious disease in which multiple immune cells are involved; among them, the liver-resident macrophages—Kupffer cells—constitute the first immunological line within hepatic sinusoids and play a central role. Dysfunction of Kupffer cells increases hepatic susceptibility to bacterial infection, thereby creating an “opportunity window” for abscess formation [8]. Using a KPLA mouse model, we observed high PD-1 expression in Kupffer cells that impaired their bactericidal capacity; PD-1 knockdown or administration of a PD-1 antibody restored this function and validated anti-PD-1 therapy as an effective intervention for KPLA. Our findings indicate that PD-1–targeted therapy represents a potential strategy against KPLA.

Hepatic macrophages, as key innate effector cells, exert dual regulatory functions in hepatic inflammation, fibrosis, and the tumor micro-environment. Kupffer cells, uniquely positioned to sample portal venous blood, efficiently capture and remove circulating pathogens, preventing systemic spread [9]. In LA models, Kupffer-cell activation and preserved bacterial-trapping activity correlate with smaller abscess volumes, implying a protective role [27]. Hypervirulent *K. pneumoniae* (HvKp) acquires genomic islands (e.g., GIE492 and ICEKp10) that facilitate translocation across the intestinal barrier and hepatic colonization [24, 28]. When the Kupffer-cell microenvironment is disrupted (e.g., in acetaminophen-induced injury), the PD-1/PD-L1 axis is up-regulated, bacterial clearance is impaired, and systemic infection risk increases [22]. Consistently, we detected notably elevated PD-1 in Kupffer cells in our KPLA model, suggesting that this up-regulation is an important mechanism underlying their reduced bactericidal activity.

PD-1, an immunoinhibitory receptor that modulates T-cell function via interaction with PD-L1/PD-L2, is critical for immune homeostasis and immune evasion. The role of the PD-1 pathway in infectious liver diseases has attracted increasing attention. In chronic hepatitis B (CHB), aberrant PD-L1 up-regulation on hepatic NK cells parallels increased PD-1 on intrahepatic T cells, jointly suppressing HBV-specific CD8^+^ T-cell responses to therapeutic vaccination [29]. In cirrhosis, monocyte PD-L1 overexpression correlates with disease severity and infection risk, whereas PD-L1 blockade reverses the immunosuppressive phenotype of hepatic macrophages and enhances their antimicrobial activity [30]. In ALF, the PD-1/PD-L1 axis exacerbates systemic immunosuppression by modulating Kupffer-cell inflammatory responses, and anti-PD-1 immunotherapy reduces infection susceptibility [22]. Aligning with these reports, we discovered that PD-1 knockdown or antibody-mediated blockade restored Kupffer-cell bacterial clearance. Importantly, PD-1 antibody administration markedly alleviated hepatic abscess in mice, indicating that anti-PD-1 therapy is a safe and effective strategy for KPLA.

In summary, this investigation reveals that PD-1-high Kupffer cells exert a critical protective function during LA pathogenesis and confirms that PD-1-based immunotherapy confers significant protective efficacy against KPLA. These findings elucidate the regulatory mechanism of the PD-1/PD-L1 pathway in liver disease, providing a theoretical basis for optimizing clinical treatment strategies. A limitation of the present work is the absence of in-depth exploration of the underlying molecular mechanisms of PD-1/PD-L1 signaling; future studies should delineate the relevant regulatory networks to identify additional therapeutic targets. Secondly, in this study, anti-PD-1 antibody administration was performed simultaneously with bacterial inoculation; therefore, the present findings mainly reveal its protective role in the early intervention of KPLA, whereas its therapeutic value remains to be validated in future studies using post-abscess-formation administration models. In addition, the current study has not yet examined survival outcomes, systemic inflammatory responses, and potential adverse effects, and these aspects will be further investigated in future work.

## Supplemental Materials

Supplementary data for this paper are available on-line only at http://jmb.or.kr.



## Figures and Tables

**Fig. 1:**
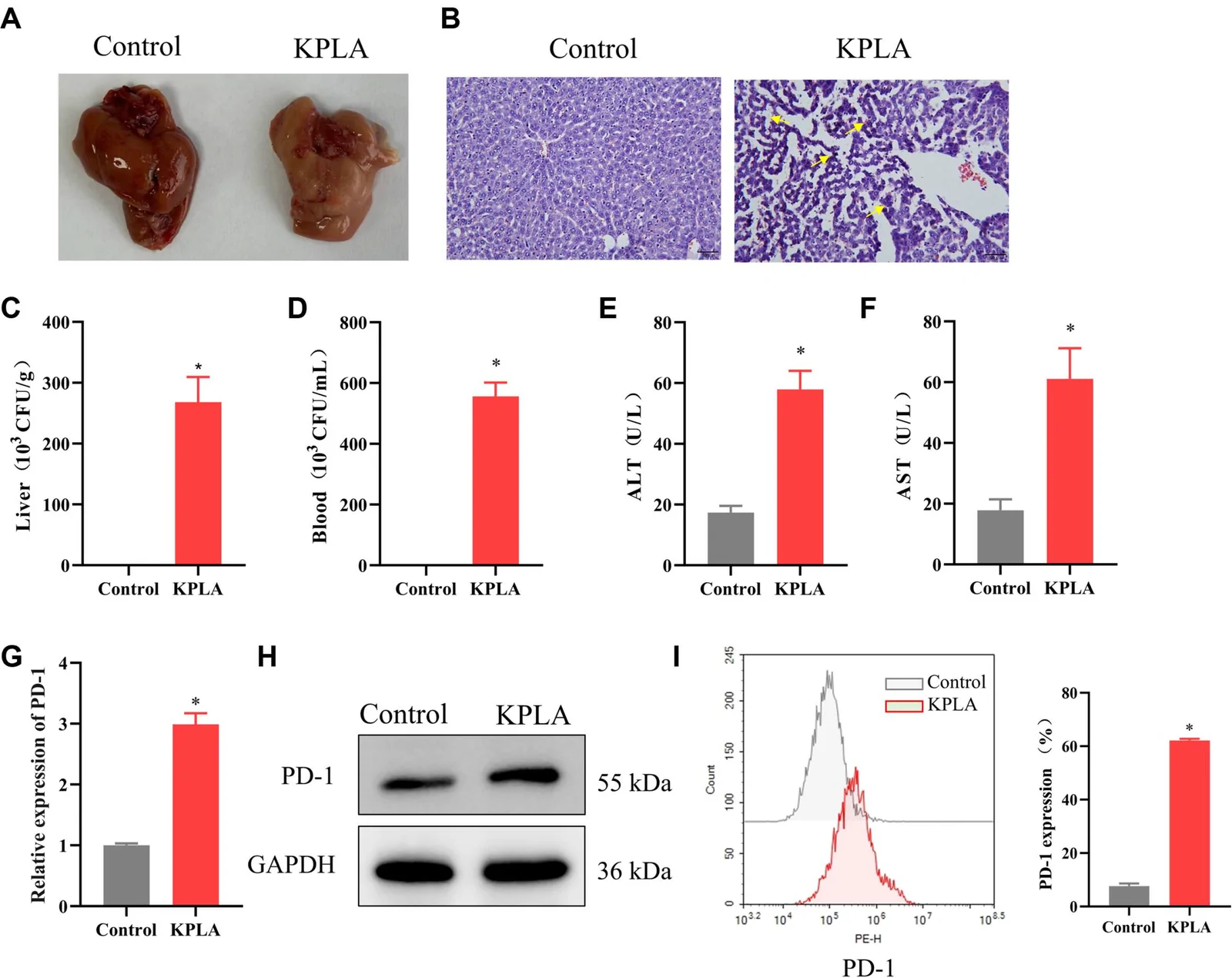
KPLA promotes PD-1 expression in Kupffer cells. (**A**) LA in mice caused by *K. pneumoniae*; (**B**) HE staining of mouse liver and microscopic observation; (**C–D**) *K. pneumoniae* burden in liver (**C**) and blood (**D**) of infected mice; (**E-F**) ELISA detection of mouse serum ALT (**E**) and AST (**F**) levels; (**FG-I**) PD-1 expression detected by RT-qPCR (**G**), Western blot (**H**), and flow cytometry (**I**). *P < 0.05 was considered statistically significant.

**Fig. 2:**
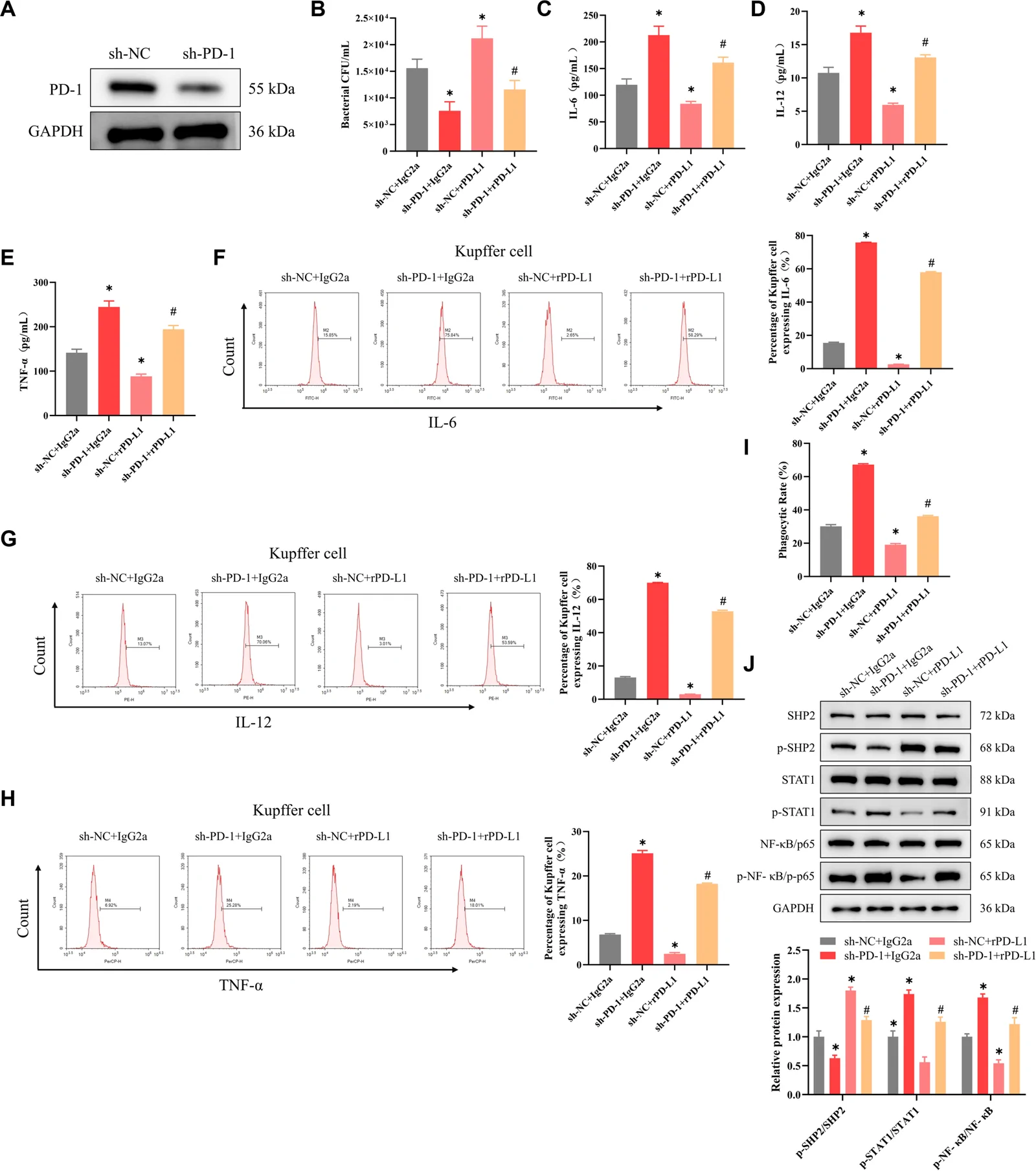
High PD-1 expression in Kupffer cells reduces bacterial clearance capacity. (**A**) Western blot detection of PD-1 protein expression; (**B**) *In vitro* bactericidal activity of Kupffer cells against *K. pneumoniae* evaluated by enumerating viable bacteria; (**C–E**) IL-6 (**C**), IL-12 (**D**), and TNF-α (**E**) levels measured by ELISA; (F-H) Flow-cytometric detection of IL-6(**F**), IL-12(**G**), and TNF-α(H) proportions; (**I**) Flow-cytometric analysis of Kupffer-cell phagocytic capacity; (**J**) Western blot detection of SHP2, p-SHP2, STAT1, p-STAT1, NF-κB p65, and p-NF-κB p65. *P < 0.05.

**Fig. 3:**
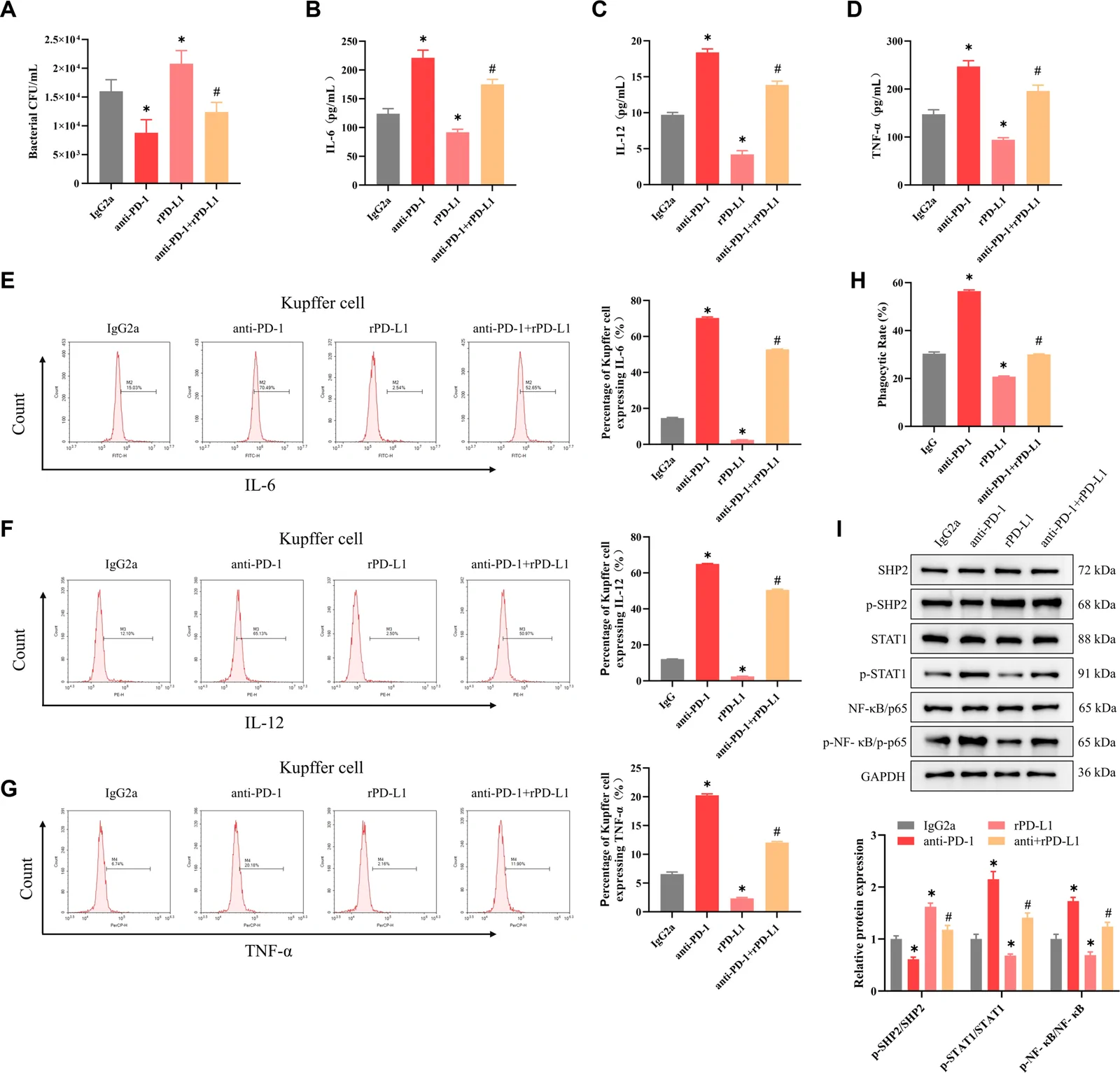
PD-1 antibody activates the bacterial clearance capacity of Kupffer cells. (**A**) *In vitro* bactericidal activity of Kupffer cells against *K. pneumoniae* evaluated by enumerating viable bacteria; (**B–D**) IL-6 (**B**), IL-12 (**C**), and TNF-α (**D**) levels measured by ELISA; (**E-G**) Flow cytometry detection of IL-6 (**E**), IL-12 (**F**), and TNF-α (**G**) proportions; (**H**) Flow-cytometric analysis of Kupffer-cell phagocytic capacity; (**I**) Western blot detection of SHP2, p-SHP2, STAT1, p-STAT1, NF-κB p65, and p-NF-κB p65. *P < 0.05.

**Fig. 4:**
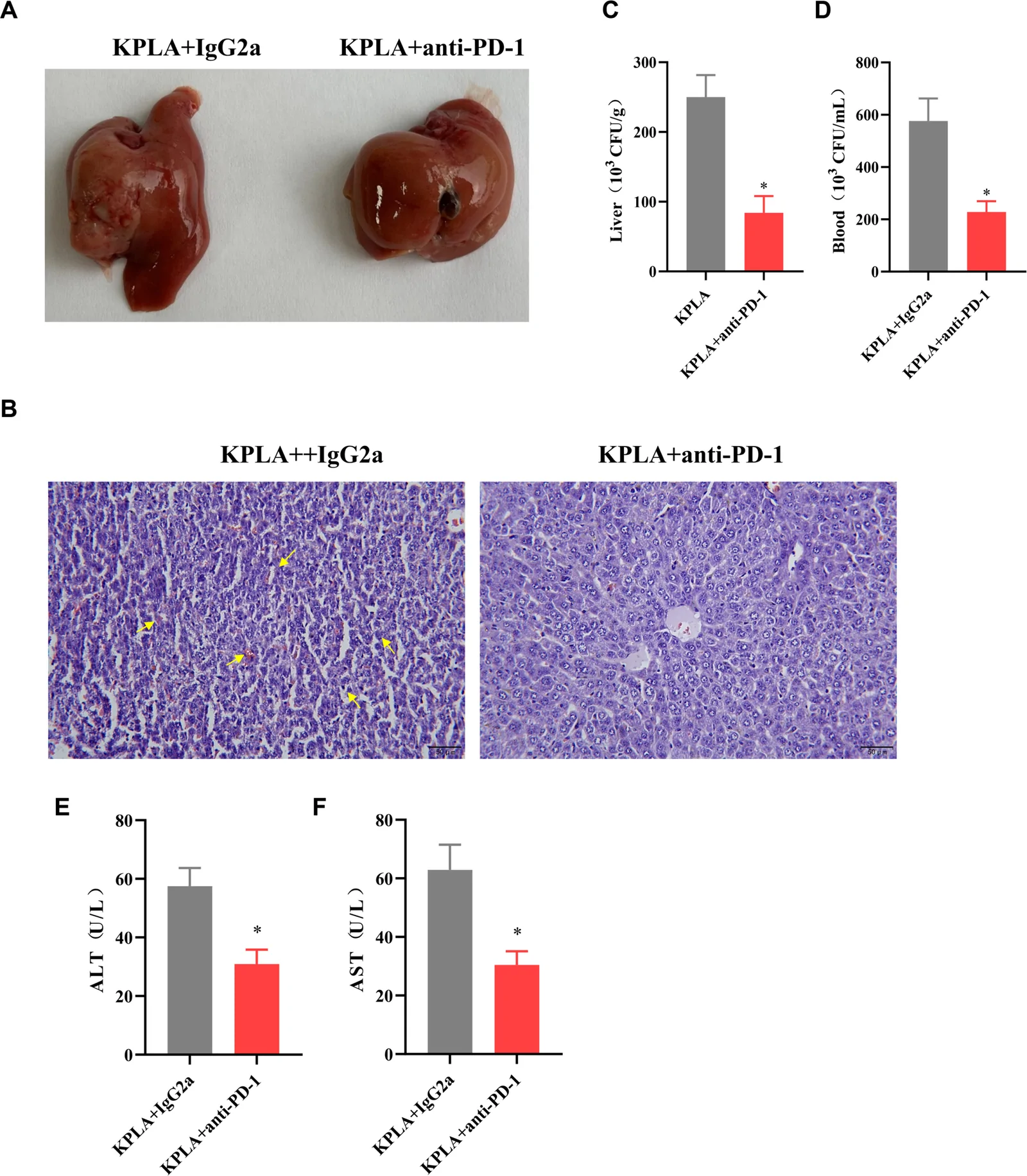
PD-1 blockade alleviates KPLA. (**A**) LA severity ameliorated after PD-1 antibody treatment; (**B**) HE staining of mouse liver and microscopic observation; (**C–D**) *K. pneumoniae* burden in liver (**C**) and blood (**D**) of infected mice. (**E–F**) ELISA detection of mouse serum ALT (**E**) and AST (**F**) levels. *P < 0.05 was considered statistically significant.

**Table 1:** The RT-qPCR primers sequences

Gene	Primer sequence (5’→3’)
PD-1	F: TTACCACAAGCTGGGAGCAG
	R: AGACATAGGCTTAGGCCCCA
GAPDH	F: GGAGAGTGTTTCCTCGTCCC
	R: ATGAAGGGGTCGTTGATGGC
