# Supplementary material for: Anti-PD-1 Therapy Alleviates Klebsiella pneumoniae-Induced Liver Abscess by Effectively Activating Kupffer Cells
Source: J Microbiol Biotechnol. 2026 Oct 19;36:e2602040. doi: 10.4014/jmb.2602.02040 (PMC13686807; doi:10.4014/jmb.2602.02040)

Kupffer cell

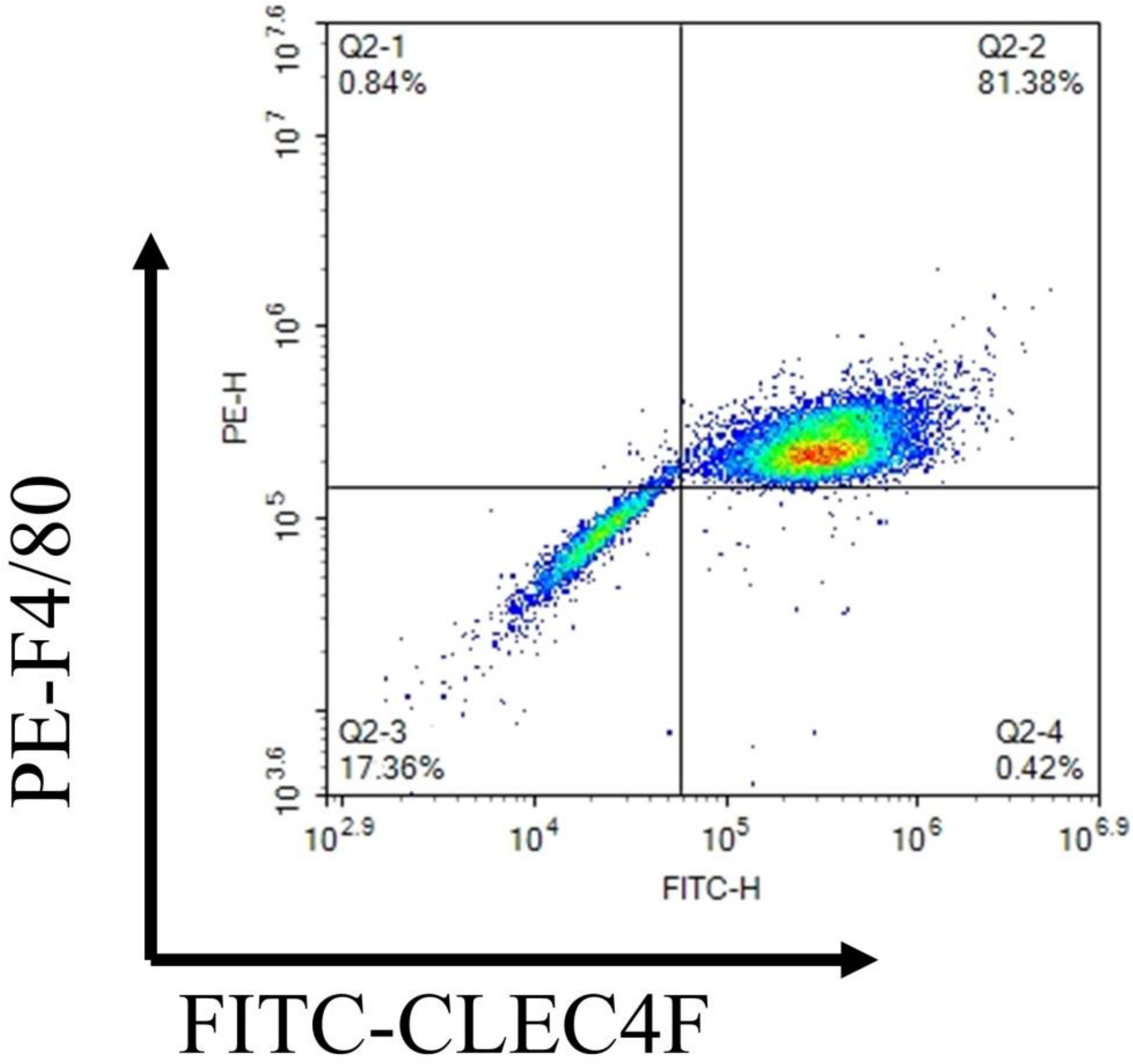

Supplement: Supplementary file 1 [file jmb-36-e2602040-supple.pdf]
